# Can local treatment prolong the sensitivity of metastatic prostate cancer to androgen deprivation or even prevent castration resistance?

**DOI:** 10.1007/s00345-020-03568-3

**Published:** 2021-01-27

**Authors:** Christina Niklas, Matthias Saar, Alessandro Nini, Johannes Linxweiler, Stefan Siemer, Kerstin Junker, Michael Stoeckle

**Affiliations:** Department of Urology and Pediatric Urology, Saarland University, Homburg/Saar, Germany

**Keywords:** Metastatic prostate cancer, Prostatectomy, Androgen deprivation treatment, Hormone-sensitivity, Castration resistance

## Abstract

**Purpose:**

A number of observational clinical studies suggest that prior primary tumor treatment favorably influences the course of metastatic prostate cancer (PCa), but its mechanisms of action are still speculative. Here, we describe the long-lasting sensitivity to various forms of androgen deprivation in patients after radical prostatectomy (RP) for locally advanced PCa as one potential mechanism.

**Methods:**

A consecutive series of 115 radical prostatectomies after inductive therapy for T4 prostate cancer was re-analyzed, and long-term survival, as well as recurrence patterns and responses to different forms of hormonal manipulation, were assessed.

**Results:**

The estimated biochemical response-free, PCa-specific, and overall survival rates after 200 months were 20%, 65%, and 47% with a median overall survival of 156 months. The majority of patients, although not cured of locally advanced PCa (84/115), showed long-term survival after RP. PCa-specific and overall survival rates of these 84 patients with biochemical recurrence were 61% and 44% at 150 months. Long-term sensitivity to ADT was found to be the main reason for the favorable tumor-specific survival in spite of biochemical recurrence.

**Conclusions:**

Sensitivity to primary or secondary hormonal manipulation was the main reason for the long-term survival of patients who had not been cured by surgery only. The results suggest that treatment of the primary tumor-bearing prostate delays castration-resistant PCa and enhances the effect of hormonal therapies in a previously unknown manner. The underlying cellular and molecular mechanisms need to be explored in more detailed analyses, which could profoundly impact treatment concepts of locally advanced and metastatic PCa.

**Supplementary Information:**

The online version contains supplementary material available at 10.1007/s00345-020-03568-3.

## Introduction

Patients with locally inoperable (T4) or primary metastatic prostate cancer (PCa) are commonly regarded as incurable. Current guidelines recommend systemic therapy such as androgen deprivation treatment (ADT) or chemotherapy combined with external beam radiotherapy to prolong survival without curation [1–3]. Emerging data suggest an additional benefit of local primary tumor treatment in the natural course of recurrent or metastatic disease [4–7]. However, no hypothesis has fundamentally been corroborated regarding how the treatment of the primary tumor could influence the course of systemic PCa.

We recently published a series of patients with cT4 PCa, defined as a fixed mass by digital rectal examination (DRE) in combination with high or very high prostate-specific antigen (PSA) levels (median 37.6 ng/ml; range 2.44–284 ng/ml) in many cases (*n* = 39 patients with PSA ≥ 50 ng/ml)[8]. These patients underwent inductive hormonal treatment until the PSA nadir, which was predictively achieved after 6–7 months. We subsequently observed a reliable clinical remission at the time of the PSA nadir, which allowed a safe removal of the prostate in almost every patient (10.3% Clavien 3, no Clavien 4/5 complications). Depending on clinical responses and the decision of the surgeon, some patients were operated on before the PSA nadir had been achieved. The vast majority of patients did not reach a non-detectable PSA after surgery, suggesting a curative effect of this approach for a minority of less than 20%. After a median follow-up of 75 (9–167) months, however, we found an unexpectedly low tumor-specific death rate of 18% [8].

To define the underlying mechanisms of this favorable outcome, we updated the clinical course of these T4 patients after radical prostatectomy (RP) in more detail, focusing on long-term survivors after proven biochemical recurrence.

## Materials and methods

Survival and treatment data of patients with initial T4 PCa, who underwent RP after inductive ADT, were updated. The operations were performed between 2000 and 2014. All patients were initially found inoperable due to a fixed tumor mass at DRE, leading to clinical stage T4. While only a few patients had an MRI or CT scan at the time of their diagnosis, the clinical impression was corroborated by transrectal ultrasound (TRUS) demonstrating advanced, non-organ-confined disease, highly elevated baseline PSA levels and/or large foci in a biopsy confirming an undifferentiated tumor. Baseline demographic data are summarized in Table 1.

**Table 1:** Patients` demographics and pathological data

Variables	Total *n =* 115
Age, yr, median (range)	66 (50–76)
PSA initial, ng/ml, median (range)	37.6 (18–61)
Gleason score (biopsy)	
≤ 7	42
> 7	60
Unknown	14
Inductive ADT duration months, median (range)	6 (2–20)
PSA preoperative, ng/ml, median (range)	0.69 (0.01–34.3)
TNM classification	
pT0-T2	22 (19.1%)
pT3	89 (77.4%)
pT4	4 (3.5%)
Positive SM	46 (40%)
LNI	38 (33%)
Positive SM and LNI	26 (22.6%)

*SM* surgical margins, *LNI* lymph node invasion

In general, inductive ADT consisted of LHRH agonists with or without antiandrogens, which was maintained until PSA nadir was reached. In addition to RP, regular lymphadenectomy was performed for the right and left external iliac region (including the obturator fossa). After the introduction of the daVinci robotic system (Intuitive Surgical Inc., Sunnyvale, CA, USA) at our center in 2006, all patients were treated with robot-assisted radical prostatectomy (RARP). Histopathologic findings of surgical specimens are summarized in Table 1.

### Statistics

Survival outcomes were analyzed with the Kaplan–Meier method and log-rank test. Statistical analysis was performed using SPSS version 23.0 (SPSS Inc., IBM Corp., Armonk, NY, USA).

## Results

**Fig. 1:**
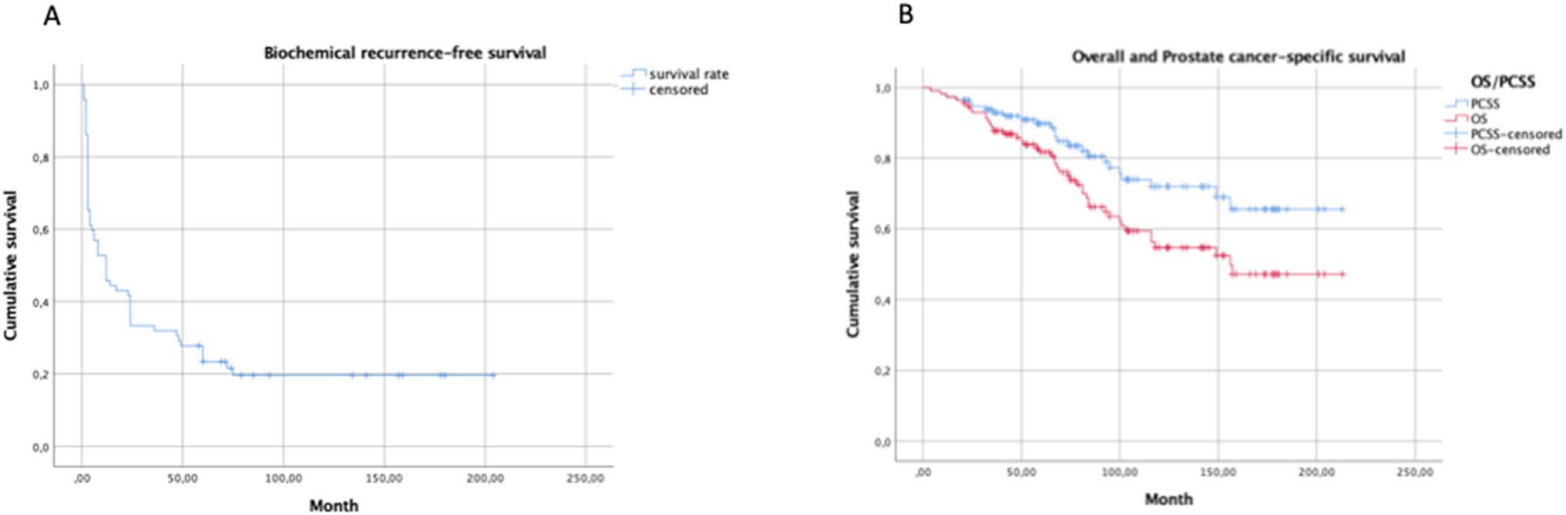
**a** Survival curve for biochemical recurrence-free survival of patients after radical prostatectomy (RP) (*n* = 72). **b** Survival curve for overall and primary metastatic prostate cancer (PCa)-specific survival of all patients after RP

**Fig. 2:**
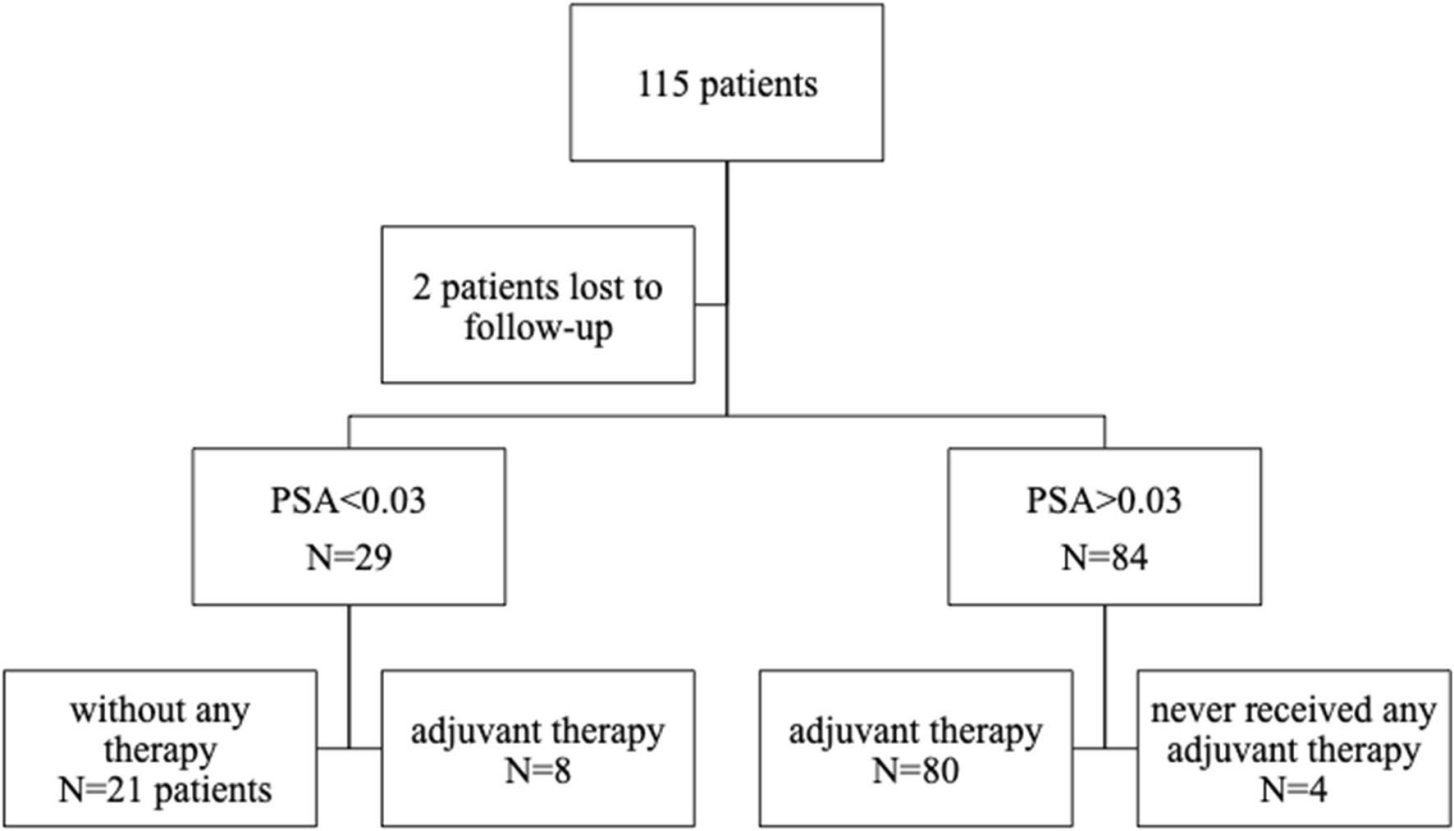
Flowchart of the total patient cohort with long-term follow-up

After a median follow-up of 81 months (inter-quartile range (IQR): 50–134), a total of 44 patients had died (38.3%) with a median time to death of 67 months (Fig. 1: OS all patients), 19 of which (43.1%) died from causes unrelated to PCa after a median time of 75 months. The estimated biochemical response-free, PCa-specific, and overall survival rates after 200 months were 20%, 65%, and 47%, respectively (Fig. 1), with a median overall survival of 156 months. Of all 115 patients, 21 patients had a complete biochemical response and never received any adjuvant therapies (Fig. 2: flow chart). Overall and PCa-specific survival rates of these 21 patients were 81% and 100% after 107 months, respectively. Postsurgical tumor characteristics of this subgroup of patients are summarized in Suppl. Table 2. Of the remaining patients, 84 had a biochemical recurrence and received further treatment. The estimated tumor-specific and overall survival rates of these 84 patients at 150 months were 61% and 44%, respectively (Suppl. Fig. 1a and b).

In total, there were 46 patients (40%) with positive surgical margin after prostatectomy, half of them underwent additional radiation therapy of the prostatic fossa during their further disease course.

Long-term data of patients with prolonged survival in spite of biochemical recurrence revealed long-term sensitivity to primary or secondary hormonal manipulation as the most important prognostic variable. In total, 47 of 84 patients with biochemical recurrence were still alive after 95 months of follow-up. Thirty-one of them received ADT with a mean biochemical response for 49 months. Only 13 patients developed castration-resistant PCa after 44 months of sensitivity to ADT alone.

We selected two informative cases to illustrate the phenomenon of ongoing hormone sensitivity (HS) for many years (Suppl. Table 1). One of these two patients had stopped adjuvant ADT five years after surgery because of a non-detectable PSA, temporarily had extremely disseminated bone metastases, and presented in moribund condition with a PSA of 8144 ng/ml. His metastases completely disappeared after the re-initiation of ADT. The total treatment time with ADT has now been more than 9 years, and PSA has returned to < 0.03 ng/ml (undetectable) 5 years after the re-start of treatment and 15 years after the initial diagnosis. Other patients, such as the second patient, developed a slowly increasing ADT-resistant PSA progression before their PSA again responded to tertiary hormonal treatment with abiraterone. These secondary complete responses persisted for more than 3 years and are ongoing.

## Discussion

The androgen sensitivity of metastatic PCa was first described by C. B. Huggins in 1941 [9]; this finding was awarded with a Nobel prize in 1966. Since then, ADT, starting as surgical castration, has remained the standard of care for metastatic and/or locally advanced PCa. Surgical castration has largely been replaced by LHRH agonist or antagonist treatment, which is regarded as being as effective as castration [10]. Unfortunately, the secondary resistance of metastatic PCa to ADT seems unavoidable. In 2002, Eisenberger and Carducci summarized that “The development of a hormone-independent state is a categorical and irreversible phenomenon observed in the majority of patients and occurs within an almost predictable time frame after the initiation of androgen deprivation” [11]. At that time, all available studies uniformly found median times to progression and tumor-related death from 12 to 18 months and 2–3 years, respectively. Survival times had not been significantly improved by the first-generation androgen receptor antagonists, such as flutamide or bicalutamide, often summarized as secondary hormonal treatment [12, 13].

During the past 15 years, however, survival times for metastatic PCa have remarkably increased. This may partly be caused by the earlier detection of metastases. This result is certainly influenced by the addition of new treatment concepts, all with a proven survival benefit of several months [14–17]. To the best of our knowledge, long-lasting or even unlimited responses to any of these treatment modalities have never been described or systematically analyzed. The Cou-AA-302 trial, which compared abiraterone plus prednisone with placebo plus prednisone in castration-resistant PCa, demonstrated a radiographic progression-free survival rate of ~ 15% after 33 months. A survival plateau of 15% or less, indicating long-term responses beyond 33 months, has not yet been published [18]. One must assume that most of the 15% of patients without radiographic progression already had rising PSA values at their last control.

Attempts to overcome castration resistance are almost as old as ADT itself. Huggins hypothesized that androgens produced by the adrenal glands induce castration resistance and he—frustraneously—tested bilateral adrenalectomy as a salvage treatment for castration-resistant PCa [19]. Meanwhile, the molecular basis of hormone resistance could at least partly be clarified on the level of the androgen receptor: modifications of the receptor allow for promiscuitive stimulation by hormones such as ACTH, TSH or even by anti-androgens such as flutamide [20, 21]. Splice variants of the receptor, such as ARV-7 [22], lack the androgen-binding domain, thus leading to permanent tumor stimulation even in the absence of any hormonal stimulus. Such splice variants are described as one main reason for cross-resistance between abiraterone and enzalutamide [22–24].

Our cases after radical prostatectomy with still elevated PSA levels (Suppl. Fig. 1) differ from all published experiences with the hormone manipulation of metastatic PCa: they have ongoing responses to various forms of hormonal treatment over time intervals that exceed all previously described time frames.

Similar phenomena have been previously observed in other settings and could be based on the same biological mechanisms. A relatively benign course for patients with PSA recurrence after RP (15 year PCa-specific 50%; Gleason ≥ 8) [25] could be caused by the long-lasting effects of delayed ADT, in a very similar manner to our results after inductive therapy [8]. Patients with recurrent mPC after prior local therapy have longer survival than comparable patients with de novo mPC [26], and this effect has been translated into modern combination therapies such as ADT plus apalutamide in the Titan study [27]. For nonmetastatic castration-resistant PCa patients, an impressively long sensitivity to apalutamide and darolutamide has been observed. Again, three-quarters of these patients had prior local therapy. Finally, patients primarily presenting with limited bone metastases seem to benefit from the addition of the treatment of their primary tumor to the standard of care [28].

Our data suggest that long-lasting hormone-dependent complete remissions of metastatic PCa occur more frequently than anticipated, predominantly in patients after local treatment. The sources that define castration resistance as an event that unavoidably occurs after 18–30 months uniformly date back to the 1990s. At that time, only a minority of patients with advanced or metastatic PCa had a prior prostatectomy or other forms of local treatment, such as radiotherapy.

Messing et al. were the first to present data in 1999 that demonstrated an impressive tumor-specific survival benefit after prostatectomy followed by immediate ADT compared to prostatectomy alone for patients affected by tumor-involved lymph nodes (pN + ; for decades, regarded as incurable) [29]. Of 47 patients randomized for combined treatment, only 17 had died, seven of which died because of PCa. Among 51 patients randomized for prostatectomy only, 28 deaths were encountered, of which 25 were PCa-related. Overall, PCa-specific and progression-free survival were significantly improved for patients randomized for prostatectomy plus ADT. Bhindi et al. retrospectively compared 158 carefully matched pN + patients treated with prostatectomy plus castration versus castration alone and found an equally impressive difference in favor of the combination therapy. The risk of dying from PCa was reduced by more than 50%, which translated into a significant improvement in 20 years of OS [30]. It, therefore, seems that the combination of prostatectomy plus ADT in this particular micrometastatic situation potentiates the effects achieved by each of the two treatment modalities alone, again corroborating the hypothesis of a modulating effect of prostate removal on the hormone sensitivity of metastatic cells. Such sensitivity was also seen in patients who underwent salvage extended pelvic lymph node dissection subsequent to PSA recurrence after radical prostatectomy [31].

Another unsolved paradox that could also be explained by the hypothesis of an ADT-modulating effect of prostatectomy is the contradictory results of radiotherapy studies that aim to treat or to prevent biochemical recurrences after prostatectomy: Irradiation significantly prevented PSA recurrences, which, however, did not translate into a survival benefit [32]. Prolonged sensitivity to ADT would equally be present both in irradiated and non-irradiated patients and could therefore counterbalance any former benefit of radiotherapy.

In principle, large amounts of data have been published that suggest a positive influence of a prior prostatectomy or other forms of local treatment on the prognosis of patients with metastatic PCa [4, 5, 33]. However, to the best of our knowledge, a hypothesis referring to a clear mode of action is still missing. The available data, in spite of scarce evidence, have fueled a discussion about the role of prostatectomy in patients with synchronous distant metastases. In analogy to kidney cancer, these operations are characterized as “cytoreductive”, for which a recent comparative case series was unable to demonstrate a survival benefit [34]. The STAMPEDE trial on the radiotherapy of the prostate in men with metastatic PCa could not find an overall survival advantage by local treatment for the complete study population but found an advantage for a subgroup defined by low metastatic burden according to the CHAARTED definition [7]. Our experience suggests that at least a minority of patients presenting with synchronous bone metastases should benefit from the removal of the primary tumor via the prevention of ADT-refractory disease. Such operations should rather be characterized as “hormone-sensitizing” instead of “cytoreductive”. However, the patient selection, role and extent of pretreatment and quantification of the therapeutic benefit undoubtedly remain to be defined by carefully designed clinical trials.

The cellular and molecular heterogeneity in the primary tumor presumably facilitates the development of therapy-resistant cell clones, which are not necessarily the origin of metastatic cells. Therefore, metastatic tumor cells by themselves may remain therapy-sensitive. We hypothesize that primary PCa including tumor cells and stroma cells or the specific prostate microenvironment influences the therapy response and plays an active role in resistance to systemic treatments and progression at metastatic sites. This concept is supported by recently published in-vitro data. The data demonstrate that the crosstalk between androgen-sensitive PCa cells and androgen-insensitive PCa cells might stimulate the progression of PCa [35]. Furthermore, therapy resistance can be induced in sensitive tumor cells by exosomes secreted by the tumor or stroma cells from resistant tumors, as demonstrated for several tumor types and therapy modalities [36].

As soon as the hypothesis of a “hormone-sensitizing” character of a prostatectomy can be proven by more robust data, the underlying molecular mechanisms will gain increased interest. Targeting these mechanisms could dramatically change PCa treatment concepts and finally fulfill Huggins’ vision: the curability of PCa at any stage of disease.

## Conclusion

Multiple data suggest a survival benefit for patients suffering from metastasized PCa by the treatment of the primary tumor-bearing prostate. The mode of action, however, has not been clarified to date. We present a patient series characterized by long-lasting or even unlimited sensitivity to various forms of hormonal treatment with a common denominator of a prior prostatectomy. This suggests that the removal or treatment of the primary tumor-bearing prostate modulates, delays, and/or even prevents resistance to ADT and can therefore lead to the chronification of metastatic PCa.

## Supplementary Information

Below is the link to the electronic supplementary material.

Supplementary file1 (DOCX 19 KB)

Supplementary file1 (DOCX 16 KB)

Supplementary file1 (DOCX 57 KB)

## References

[1] Cornford P, Bellmunt J, Bolla M, Briers E, De Santis M, Gross T, Henry AM, Joniau S, Lam TB, Mason MD, van der Poel HG, van der Kwast TH, Rouviere O, Wiegel T, Mottet N (2017). EAU-ESTRO-SIOG Guidelines on prostate cancer part II: treatment of relapsing, metastatic, and castration-resistant prostate cancer. Eur Urol.

[2] Mohler JL, Armstrong AJ, Bahnson RR, D'Amico AV, Davis BJ, Eastham JA, Enke CA, Farrington TA, Higano CS, Horwitz EM, Hurwitz M, Kane CJ, Kawachi MH, Kuettel M, Lee RJ, Meeks JJ, Penson DF, Plimack ER, Pow-Sang JM, Raben D, Richey S, Roach M, Rosenfeld S, Schaeffer E, Skolarus TA, Small EJ, Sonpavde G, Srinivas S, Strope SA, Tward J, Shead DA, Freedman-Cass DA (2016). Prostate cancer, Version 1.2016. J Nat Comprehen Cancer Netw.

[3] Ranasinghe WKB, Reichard CA, Bathala T, Chapin BF (2019). Management of cT4 prostate cancer. Eur Urol Focus.

[4] Rusthoven CG, Jones BL, Flaig TW, Crawford ED, Koshy M, Sher DJ, Mahmood U, Chen RC, Chapin BF, Kavanagh BD, Pugh TJ (2016). Improved survival with prostate radiation in addition to androgen deprivation therapy for men with newly diagnosed metastatic prostate cancer. J Clin Oncol.

[5] Culp SH, Schellhammer PF, Williams MB (2014). Might men diagnosed with metastatic prostate cancer benefit from definitive treatment of the primary tumor? A SEER-based study. Eur Urol.

[6] Gratzke C, Engel J, Stief CG (2014). Role of radical prostatectomy in metastatic prostate cancer: data from the Munich Cancer Registry. Eur Urol.

[7] Parker CC, James ND, Brawley CD, Clarke NW, Hoyle AP, Ali A, Ritchie AWS, Attard G, Chowdhury S, Cross W, Dearnaley DP, Gillessen S, Gilson C, Jones RJ, Langley RE, Malik ZI, Mason MD, Matheson D, Millman R, Russell JM, Thalmann GN, Amos CL, Alonzi R, Bahl A, Birtle A, Din O, Douis H, Eswar C, Gale J, Gannon MR, Jonnada S, Khaksar S, Lester JF, O'Sullivan JM, Parikh OA, Pedley ID, Pudney DM, Sheehan DJ, Srihari NN, Tran ATH, Parmar MKB, Sydes MR (2018). Radiotherapy to the primary tumour for newly diagnosed, metastatic prostate cancer (STAMPEDE): a randomised controlled phase 3 trial. The Lancet.

[8] Hajili T, Ohlmann CH, Linxweiler J, Niklas C, Janssen M, Siemer S, Stoeckle M, Saar M (2019). Radical prostatectomy in T4 prostate cancer after inductive androgen deprivation: results of a single-institution series with long-term follow-up. BJU Int.

[9] Huggins C, Hodges CV (1941). Studies on prostatic cancer. I. The effect of castration, of estrogen and of androgen injection on serum phosphatases in metastatic carcinoma of the prostate. Can Res.

[10] Kaisary AV, Tyrrell CJ, Peeling WB, Griffiths K (1991). Comparison of LHRH analogue (Zoladex) with orchiectomy in patients with metastatic prostatic carcinoma. Br J Urol.

[11] Eisenberger MA, Carducci MA, Walsh PC, Retik AB, Vaughan ED (2002). Chemotherapy For Hormone-Resistant Prostate Cancer. Campbell's Urology.

[12] Robinson MR, Smith PH, Richards B, Newling DW, de Pauw M, Sylvester R (1995). The final analysis of the EORTC Genito-Urinary Tract Cancer Co-Operative Group phase III clinical trial (protocol 30805) comparing orchidectomy, orchidectomy plus cyproterone acetate and low dose stilboestrol in the management of metastatic carcinoma of the prostate. Eur Urol.

[13] Maximum androgen blockade in advanced prostate cancer: an overview of the randomised trials. Prostate Cancer Trialists' Collaborative Group (2000) Lancet (London, England) 355 (9214):1491–1498 10801170

[14] Tannock IF, de Wit R, Berry WR, Horti J, Pluzanska A, Chi KN, Oudard S, Theodore C, James ND, Turesson I, Rosenthal MA, Eisenberger MA (2004). Docetaxel plus prednisone or mitoxantrone plus prednisone for advanced prostate cancer. N Engl J Med.

[15] Ryan CJ, Smith MR, de Bono JS, Molina A, Logothetis CJ, de Souza P, Fizazi K, Mainwaring P, Piulats JM, Ng S, Carles J, Mulders PF, Basch E, Small EJ, Saad F, Schrijvers D, Van Poppel H, Mukherjee SD, Suttmann H, Gerritsen WR, Flaig TW, George DJ, Yu EY, Efstathiou E, Pantuck A, Winquist E, Higano CS, Taplin ME, Park Y, Kheoh T, Griffin T, Scher HI, Rathkopf DE (2013). Abiraterone in metastatic prostate cancer without previous chemotherapy. N Engl J Med.

[16] Parker C, Nilsson S, Heinrich D, Helle SI, O'Sullivan JM, Fossa SD, Chodacki A, Wiechno P, Logue J, Seke M, Widmark A, Johannessen DC, Hoskin P, Bottomley D, James ND, Solberg A, Syndikus I, Kliment J, Wedel S, Boehmer S, Dall'Oglio M, Franzen L, Coleman R, Vogelzang NJ, O'Bryan-Tear CG, Staudacher K, Garcia-Vargas J, Shan M, Bruland OS, Sartor O (2013). Alpha emitter radium-223 and survival in metastatic prostate cancer. N Engl J Med.

[17] Beer TM, Armstrong AJ, Rathkopf DE, Loriot Y, Sternberg CN, Higano CS, Iversen P, Bhattacharya S, Carles J, Chowdhury S, Davis ID, de Bono JS, Evans CP, Fizazi K, Joshua AM, Kim CS, Kimura G, Mainwaring P, Mansbach H, Miller K, Noonberg SB, Perabo F, Phung D, Saad F, Scher HI, Taplin ME, Venner PM, Tombal B (2014). Enzalutamide in metastatic prostate cancer before chemotherapy. N Engl J Med.

[18] Rathkopf DE, Smith MR, de Bono JS, Logothetis CJ, Shore ND, de Souza P, Fizazi K, Mulders PF, Mainwaring P, Hainsworth JD, Beer TM, North S, Fradet Y, Van Poppel H, Carles J, Flaig TW, Efstathiou E, Yu EY, Higano CS, Taplin ME, Griffin TW, Todd MB, Yu MK, Park YC, Kheoh T, Small EJ, Scher HI, Molina A, Ryan CJ, Saad F (2014). Updated interim efficacy analysis and long-term safety of abiraterone acetate in metastatic castration-resistant prostate cancer patients without prior chemotherapy (COU-AA-302). Eur Urol.

[19] Huggins C, Scott WW (1945). Bilateral adrenalectomy in prostatic cancer. Ann Surg.

[20] Feldman BJ, Feldman D (2001). The development of androgen-independent prostate cancer. Nat Rev Cancer.

[21] Yuan X, Balk SP (2009). Mechanisms mediating androgen receptor reactivation after castration. Urol Oncol.

[22] Antonarakis ES, Lu C, Wang H, Luber B, Nakazawa M, Roeser JC, Chen Y, Mohammad TA, Chen Y, Fedor HL, Lotan TL, Zheng Q, De Marzo AM, Isaacs JT, Isaacs WB, Nadal R, Paller CJ, Denmeade SR, Carducci MA, Eisenberger MA, Luo J (2014). AR-V7 and resistance to enzalutamide and abiraterone in prostate cancer. N Engl J Med.

[23] Lombard AP, Liu L, Cucchiara V, Liu C, Armstrong CM, Zhao R, Yang JC, Lou W, Evans CP, Gao AC (2018). Intra vs inter cross-resistance determines treatment sequence between taxane and AR-targeting therapies in advanced prostate cancer. Mol Cancer Ther.

[24] Schrader AJ, Boegemann M, Ohlmann CH, Schnoeller TJ, Krabbe LM, Hajili T, Jentzmik F, Stoeckle M, Schrader M, Herrmann E, Cronauer MV (2014). Enzalutamide in castration-resistant prostate cancer patients progressing after docetaxel and abiraterone. Eur Urol.

[25] Freedland SJ, Humphreys EB, Mangold LA, Eisenberger M, Dorey FJ, Walsh PC, Partin AW (2005). Risk of prostate cancer-specific mortality following biochemical recurrence after radical prostatectomy. JAMA.

[26] Gravis G, Boher JM, Chen YH, Liu G, Fizazi K, Carducci MA, Oudard S, Joly F, Jarrard DM, Soulie M, Eisenberger MJ, Habibian M, Dreicer R, Garcia JA, Hussain MHM, Kohli M, Vogelzang NJ, Picus J, DiPaola R, Sweeney C (2018). Burden of metastatic castrate naive prostate cancer patients, to identify men more likely to benefit from early Docetaxel: further analyses of CHAARTED and GETUG-AFU15 studies. Eur Urol.

[27] Bjartell AS, Ye D, Agarwal N, Chung BH, Given R, Merseburger A, Özgüroğlu M, Juárez Soto A, Uemura H, Lopez-Gitlitz A, Li G, Mc Carthy S, Chi KN, Chowdhury S (2020). Apalutamide (APA) for metastatic castration-sensitive prostate cancer (mCSPC) in TITAN: outcomes in patients (pts) with de novo (D1) mCSPC vs. progression to mCSPC after localized disease (D0) at diagnosis. Eur Urol Open Sci.

[28] Burdett S, Boeve LM, Ingleby FC, Fisher DJ, Rydzewska LH, Vale CL, van Andel G, Clarke NW, Hulshof MC, James ND, Parker CC, Parmar MK, Sweeney CJ, Sydes MR, Tombal B, Verhagen PC, Tierney JF, Collaborators SMR (2019). Prostate radiotherapy for metastatic hormone-sensitive prostate cancer: A STOPCAP systematic review and meta-analysis. Eur Urol.

[29] Messing EM, Manola J, Sarosdy M, Wilding G, Crawford ED, Trump D (1999). Immediate hormonal therapy compared with observation after radical prostatectomy and pelvic lymphadenectomy in men with node-positive prostate cancer. N Engl J Med.

[30] Bhindi B, Rangel LJ, Mason RJ, Gettman MT, Frank I, Kwon ED, Tollefson MK, Thompson RH, Boorjian SA, Karnes RJ (2017). Impact of radical prostatectomy on long-term oncologic outcomes in a matched cohort of men with pathological node positive prostate cancer managed by castration. J Urol.

[31] Osmonov DK, Aksenov AV, Trick D, Naumann CM, Hamann MF, Faddan AA, Junemann KP (2016). Cancer-specific and overall survival in patients with recurrent prostate cancer who underwent salvage extended pelvic lymph node dissection. BMC Urol.

[32] Bolla M, van Poppel H, Tombal B, Vekemans K, Da Pozzo L, de Reijke TM, Verbaeys A, Bosset JF, van Velthoven R, Colombel M, van de Beek C, Verhagen P, van den Bergh A, Sternberg C, Gasser T, van Tienhoven G, Scalliet P, Haustermans K, Collette L (2012). Postoperative radiotherapy after radical prostatectomy for high-risk prostate cancer: long-term results of a randomised controlled trial (EORTC trial 22911). Lancet (London, England).

[33] Sooriakumaran P, Nyberg T, Akre O, Widmark A, Hamdy F, Graefen M, Carlsson S, Steineck G, Wiklund NP (2017). Survival among men at high risk of disseminated prostate cancer receiving initial locally directed radical treatment or initial androgen deprivation therapy. Eur Urol.

[34] Steuber T, Berg KD, Roder MA, Brasso K, Iversen P, Huland H, Tiebel A, Schlomm T, Haese A, Salomon G, Budaus L, Tilki D, Heinzer H, Graefen M, Mandel P (2017). Does cytoreductive prostatectomy really have an impact on prognosis in prostate cancer patients with low-volume bone metastasis?.

[35] Takezawa Y, Izumi K, Machioka K, Iwamoto H, Naito R, Makino T, Kadomoto S, Natsagdorj A, Kadono Y, Keller ET, Zhang J, Mizokami A (2018). Crosstalk between androgen-sensitive and androgen-insensitive prostate cancer cells. Anticancer Res.

[36] Bach DH, Hong JY, Park HJ, Lee SK (2017). The role of exosomes and miRNAs in drug-resistance of cancer cells. Int J Cancer.

