# Supplementary material for: Can local treatment prolong the sensitivity of metastatic prostate cancer to androgen deprivation or even prevent castration resistance?
Source: World J Urol. 2021 Jan 27;39(9):3231–7. doi: 10.1007/s00345-020-03568-3 (PMC8510934; doi:10.1007/s00345-020-03568-3)
Supplement: Supplementary file 1 — Supplementary file1 (DOCX 19 KB) [file 345_2020_3568_MOESM1_ESM.docx]

**Suppl. table 1 - Medical history of two informative cases**

|  | **Patient No 1** | **Patient No 2** |
| --- | --- | --- |
| **Age *(yrs)*/date/PSA at diagnosis *(ng/ml)*** | 66 / Nov 2004 / 299 | 60 / Dec 2005 / 48 |
| **Duration of inductive treatment *(months)*** | 8 | 2 |
| **Type of inductive treatment** | LHRH-analogon | Cyproteronacetate |
| **Date of radical prostatectomy** | July 2005 | Jan 2006 |
| **PSA at RPE *(ng/ml)*** | 0.67 | 3.8 |
| **Gleason-Score of biopsy specimen** | 9 | 7b |
| **pTNM/SM** | pT3a N0 cM0 PSM | pT3b N1 (5/14) cM0 NSM |
| **Postop. PSA *(ng/ml)*** | <0.03 | 0.76 |
| **Immediate continuation of ADT after radical prostatectomy** | yes | yes |
| **Postop. ADT monotherapy** | July 2006 - Nov 2010  Ongoing since Sept 2015 | July 2006 - Mar 2015 |
| **PSA at time of termination of/**  **progression from ADT *(ng/ml)* and date** | <0.03 (Nov. 2010) | 20.59 (Mar 2015) |
| **Type of treatments other than ADT/date and reason of their initiation**  **With PSA at initiation of treatment and nadir during treatment *(ng/ml)*** | none | 1. **Imatinib** for one month/June 2006 *(study treatment)* because of early biochemical recurrence after RPE  2. **Abiraterone** at PSA 20.59/Mar 2015/PSA-nadir <0.03 (since Aug 2015) *(study treatment Mar 2015 - Nov 2017; continuation since Nov 2017 until now)* |
| **Location/Manifestation of metastastes/date of manifestation** | Massive bone metastases/Sept 2015 | Lymph node metastases/Dec 2014  Local recurrence rectal wall/Feb 2015 |
| **Maximum PSA after interruption of ADT/manifestation of CRPC *(ng/ml)*** | 8144 | 20.59 |
| **PSA at /date of re-initiation of ADT** | 8144/Sept 2015 | n.a. |
| **Intermittent ADT** | no | no |
| **Survival status** | alive | alive |
| **Survival after initial diagnosis *(yrs)*** | 16 | 15 |
| **Date of last follow-up** | Aug 2020 | July 2020 |
| **Current treatment regimen *at last control*** | LHRH-analogon | Abiraterone |
| **PSA (ng/ml) at last control** | <0.03 | <0.03 |
