# Supplementary material for: Can local treatment prolong the sensitivity of metastatic prostate cancer to androgen deprivation or even prevent castration resistance?
Source: World J Urol. 2021 Jan 27;39(9):3231–7. doi: 10.1007/s00345-020-03568-3 (PMC8510934; doi:10.1007/s00345-020-03568-3)
Supplement: Supplementary file 2 — Supplementary file1 (DOCX 16 KB) [file 345_2020_3568_MOESM2_ESM.docx]

**Suppl. table 2 - Postsurgical tumor characteristics of patients with complete biochemical response without any adjuvant therapies**

| **Variables** | **Patients**  **n=21** |
| --- | --- |
| **Gleason score (biopsy)**  **≤7**  **>7**  **Unknown** | 6  11  4 |
| **TNM classification** |  |
| **pT0-T2** | 8 (38.4%) |
| **pT3** | 12 (57.1%) |
| **pT4** | 1 (4.8%) |
| **Positive SM** | 6 (28.6%) |
| **LNI** | 3 (14.3%) |
|  |  |
| SM, surgical margins  LNI, lymph node invasion |  |
