# Supplementary figures and images for: Can local treatment prolong the sensitivity of metastatic prostate cancer to androgen deprivation or even prevent castration resistance?
Source: World J Urol. 2021 Jan 27;39(9):3231–7. doi: 10.1007/s00345-020-03568-3 (PMC8510934; doi:10.1007/s00345-020-03568-3)

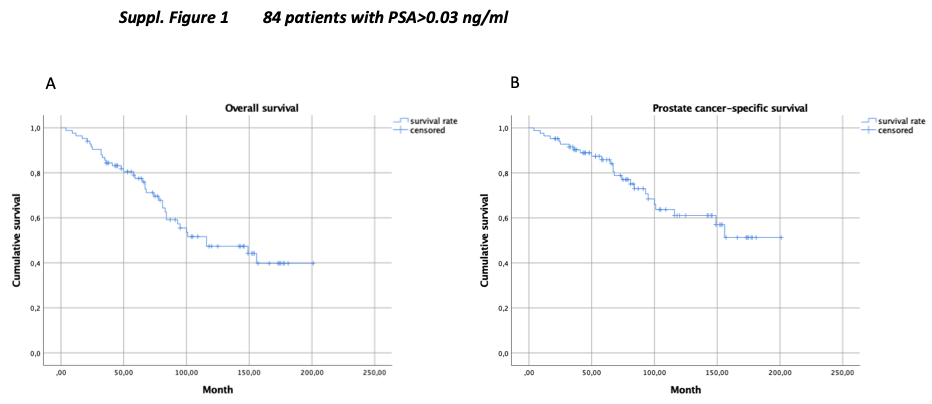

Supplement: Supplementary file 3 — Supplementary file1 (DOCX 57 KB) [file 345_2020_3568_MOESM3_ESM.docx]
